# Automatic Detection and Classification of Hypertensive Retinopathy with Improved Convolution Neural Network and Improved SVM

**DOI:** 10.3390/bioengineering11010056

**Published:** 2024-01-05

**Authors:** Usharani Bhimavarapu, Nalini Chintalapudi, Gopi Battineni

**Affiliations:** 1 Department of Computer Science and Engineering, Koneru Lakshmaiah Education Foundation, Vaddeswaram 522302, India; 2 Clinical Research Centre, School of Medicinal and Health Products Sciences, University of Camerino, 62032 Camerino, Italy; nalini.chintalapudi@unicam.it

**Keywords:** channel attention, spatial attention, spatial pooling, pooling, SVM classifier, aKNN classifier

## Abstract

Hypertensive retinopathy (HR) results from the microvascular retinal changes triggered by hypertension, which is the most common leading cause of preventable blindness worldwide. Therefore, it is necessary to develop an automated system for HR detection and evaluation using retinal images. We aimed to propose an automated approach to identify and categorize the various degrees of HR severity. A new network called the spatial convolution module (SCM) combines cross-channel and spatial information, and the convolution operations extract helpful features. The present model is evaluated using publicly accessible datasets ODIR, INSPIREVR, and VICAVR. We applied the augmentation to artificially increase the dataset of 1200 fundus images. The different HR severity levels of normal, mild, moderate, severe, and malignant are finally classified with the reduced time when compared to the existing models because in the proposed model, convolutional layers run only once on the input fundus images, which leads to a speedup and reduces the processing time in detecting the abnormalities in the vascular structure. According to the findings, the improved SVM had the highest detection and classification accuracy rate in the vessel classification with an accuracy of 98.99% and completed the task in 160.4 s. The ten-fold classification achieved the highest accuracy of 98.99%, i.e., 0.27 higher than the five-fold classification accuracy and the improved KNN classifier achieved an accuracy of 98.72%. When computation efficiency is a priority, the proposed model’s ability to quickly recognize different HR severity levels is significant.

## 1. Introduction

Blood pressure is a measurement of the pressure on artery walls caused by blood circulation and elevates hypertension. Hypertension is the cause of various cardiovascular diseases (CVD), which affect the heart and blood vessels. It is reported that more than 35.5% of patients know the side effects of hypertension and the treatments that accompany them [1]. Severe target organ damage and other complications are not the only causes of death in hypertensive patients [2,3,4,5]. As a result of systemic changes brought on by hypertension, hypertensive retinopathy (HR) can have a significant negative impact on the retina [6]. At the earlier stages, HR does not show any symptoms. More than 90% of HR patients can regain their vision or prevent the problem from affecting their eyesight if the proper treatment is taken at the appropriate time [7]. Regular eye exams are the only approach to identifying this condition. 

The retinal examination is performed using color fundus images obtained by fundus photography [8]. Experts analyze these images to recognize possible HR defects [9]. However, it is challenging to guarantee that all patients receive frequent exams, given the rising number of HR cases. Undiagnosed cases of hypertension are increasing day by day, and manual diagnosis is insufficient for treating them all. A constant checkup for all hypertensive patients is simply insufficient, which suggests the importance of finding alternative ways to diagnose HR [10]. With automated HR detection, human error can be reduced, and ophthalmologists can extract even minute lesions that are difficult to correctly identify with manual HR detection. In addition to improving the overall performance of HR detection, the computer-aided diagnosis (CAD) system allows ophthalmologists to detect abnormalities at an early stage [11]. Through the interpretation of fundus images, CAD systems improve HR diagnosis and reduce computational complexity [12]. 

The most significant barrier to HR detection lies in separating small-sized arteries and veins through the extraction of characteristic features from the fundus image. The pre-processed fundus image can provide differentiating features to diagnose and categorize HR. Building features based on statistics, color, intensity, shape, structure, and texture can be achieved by creating a master feature vector [13,14,15,16,17]. Deep learning models extract the discriminative features from the training data without manual intervention and then classify the test data according to multiple grades. The primary difficulty in HR detection is extracting distinguishing features from fundus images, which can be enhanced by utilizing improved deep learning models [18].

A hybrid model was proposed to detect red spots using color equalization, CLAHE-based contrast improvement, hand-crafted features, and convolutional neural networks (CNN) features [19]. To classify lesions, a random forest classifier was used, and the results mentioned that it achieved 93% accuracy. A study suggested a model for segmenting retinal arteries that used area growth and level-set approaches [20]. In [21], the authors used distinct machine learning (ML) models for the classification of k-means clustering. Histograms and wavelet grayscale co-occurrence run length matrices were utilized by them in extracting features from the segmented image that occurred with 98.83% accuracy. Another study proposed a pool-less residual segmentation network to diagnose high blood pressure retinopathy and achieved 82% sensitivity [22]. Alternatively, the depth-wise separable CNN detects HR from fundus images and achieves an accuracy of 95% [23].

In the majority of existing models, extensive CNNs are continually applied to the raw pixels of thousands of affected areas in each image, which is computationally time-consuming. Feature extraction is used to overcome the limitations of the previous models by utilizing spatial pooling on the feature maps. The proposed method inherits highly detailed CNN feature maps and the flexibility of improved spatial pooling on a range of sizes, which yields outstanding accuracy and effectiveness. The flexibility of the spatial pooling allows us to train on fundus images of different sizes or scales. Scale invariance increases and overfitting decreases during training with various sized fundus images. The proposed model computes features more quickly and accurately than the current traditional methods.

### Research Contributions

1. The convolutional layers run only once on the full fundus image, which leads to a speedup and reduces the processing time to detect abnormalities in the vascular structure;
2. Extracted 24 features and classified the vessels of the retinal fundus images;
3. Retina vessel segmentation with the proposed model on multiple data sets. The proposed model boosts the performance, resulting in an increase in the segmentation accuracy of the blood vessels.

## 2. Methods and Materials

**Figure 1:**
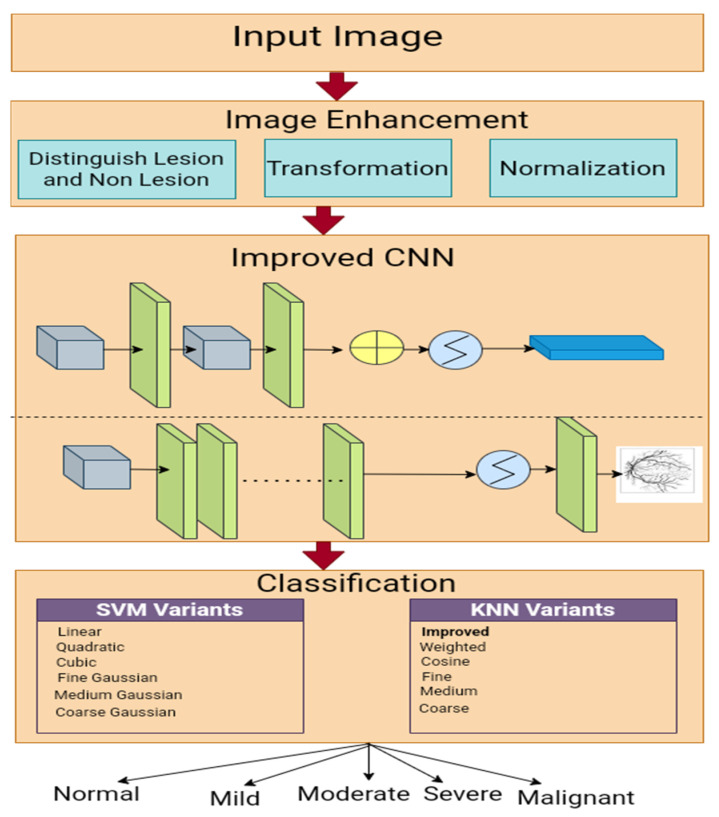
An experimental framework for HR classification.

We employed an enhanced Gaussian distribution to enhance the fundus images, improved CNN-based segmentation, and a multi-class SVM classifier to grade the HR. The workflow used in this study is presented in Figure 1.

### 2.1. Image Collection

We collected fundus images from publicly available datasets ODIR [24], INSPIREVR [25], and VICAVR [26]. Additionally, 40 images from INSPIREVR, 58 from VICAVR, and 107 fundus from the ODIR dataset. INSPIREVR fundus images have a resolution of 2392 × 2048 pixels and 768 × 584 resolution for the VICAVR database. According to the worldwide categorization standards for HR, these images are categorized by trained graders into six classes (i.e., normal, mild, moderate, severe, malignant, and ungradable) [27]. Table 1 tabulates the symptoms that exist for the five HR classes. Retinal images were classified as ungradable if the quality of the key regions was insufficient to allow for a confident grading, or if the region was only partially visible due to obstructions such as dark shadows or artifacts. We excluded the retina images if the fundus image had any other associated retinal disease except HR and we termed these types of images as ungradable images. We applied the augmentation techniques like random rotation, shearing, and translating to the collected dataset as it collected set consists of a small sample size. The final distribution of the dataset was normal (200), mild (400), moderate (200), severe (200), and malignant (200).The final dataset consists of 1200 fundus images and we split the dataset into 80:20 ratio where 80% are used for the training set and 20% are used for the testing set, i.e., 960 images for training and 240 fundus images for testing.

### 2.2. Pre-Processing

Direct segmentation utilizing the original image produces poor results and ambiguous vessel boundaries because of the peculiarities of the retinal fundus vessel images and their acquisition. As a result, before segmentation, pre-processing must be performed to improve the vessel information. The term “vascular network” refers to the network of vessels that make up the retina; these vessels are made up of arteries and veins. The containers include branches and roots, much like trees. These vessels have a tabular shape with gradually changing widths and orientations. It is challenging to see the vessels due to the low and inconsistent contrast caused by the differences in the vessels. To improve and align the retinal vessels for the retinal segmentation procedure, pre-processing measures are required. To provide a well-contrasted image, we employ contrast enhancement techniques in our pre-processing processes for the retinal fundus image. Retinal color fundus images with low and variable contrast and uneven lighting can be corrected by raising the contrast level of each channel to produce a well-contrast image. Red, green, and blue, often known as RGB channels of retinal color backdrop images, are the three channels of fundus images. Each channel has unique imaging characteristics. While the green channel has less noisy pixels and provides higher contrast, the blue channel has more noise and shadow. The red channel is brighter and contains noise. The process of processing color images adds extraneous information, which makes processing data for any segmentation or classification approach more difficult. There is noise inside the vessel and in the surrounding boundaries, an improved Gaussian process should be used to denoise this.

This study proposed the enhanced Gaussian distribution to improve the lesions’ visual contents. Gaussian distribution with a probability value enhanced the image contrast, followed by an HSV transformation. Consider D as the set of captured images and O (x, y) as the original RGB image with dimensions (300 × 250). Gaussian distributions can be used to improve low-contrast images [28]. Mathematically, it is written as

(1) $$G=\frac{1}{\sqrt{2\Pi S}}e^{-}{\left(\frac{o_{xy}-m}{s}\right)}^{2}$$

where s is the standard deviation, s = $\sqrt{\frac{{\sum o}_{xy}^{2}}{n}-{\left(\frac{{\sum o}_{xy}}{n}\right)}^{2}}$, m is the mean where m = $\sum_{x,y=1}^{n}\frac{o_{xy}}{n}$, o is the input image, and n is the sample count.

To distinguish between lesion and non-lesion, we used the following formula

(2) $$\frac{1+f}{g}$$

where f = $\sum_{x,y=1}^{n}p(\frac{{1-o}_{xy}}{n})$ and F represents the unique value and g = $\frac{1+G}{\lambda }$ and g is the scaling parameter, p represents the probability, n is the pixels count, and λ is the sampling parameter with a 0.2 value. In the follow-up of the lesion and non-lesion differentiation, HSV transformation was applied and mathematically it is presented as

(3) $$\beta_{h}=\;\cos^{-1}\frac{\left(\frac{1}{2}(2\beta_{r}-\beta_{g}-\beta_{b})\right)}{{\left(\beta_{r}-\beta_{g}\right)}^{2}\left(\beta_{r}-\beta_{b}\right)\left(\beta_{g}-\beta_{b}\right)}$$

(4) $$\beta_{s}=\frac{\max\left(\beta_{r},\beta_{g},\beta_{b}\right)-\min\left(\beta_{r},\beta_{g},\beta_{b}\right)}{\max\left(\beta_{r},\beta_{g},\beta_{b}\right)}$$

(5) $$\beta_{v}=\max\left(\beta_{r},\beta_{g},\beta_{b}\right)$$

here, $\beta_{r},\beta_{g},\beta_{b}$ denotes the RGB channels, and each is formulated as

(6) $$\beta_{r}=\frac{r}{r+g+b},\;\beta_{g}=\frac{g}{r+g+b}\;\mathrm{and}\;\beta_{b}=\frac{b}{r+g+b}$$

Normalize the pixel values using Doane’s formulae and the equations are presented as

(7) $$\psi_{h}=1+\log_{2}(\beta_{h})+\log_{2}(k)$$

here, $\psi_{h}$ represents the does function, where k represents the skewness and it is represented as

(8) $$k=\frac{f\left(\beta_{h}^{3}\right)-3mf\left(\beta_{h}^{2}\right)+m^{2}f\left(\beta_{h}\right)-m^{3}}{s^{3}}$$

The probability of each channel is computed as (x, y) = $\frac{\beta_{xy}}{n}$; here, β_xy_ is the channel’s favorable pixels and n is the total number of pixels.

As a result of averaging the probabilities of three channels, using the threshold function, it was possible to identify the most suitable channel presented as

(9) $$\beta_{P}\left(c,d\right)=\left\{\begin{array}{c} \beta_{h}\;\mathrm{if}\;{f(\beta }_{h}(c,d))<m_{p(c,d)}\\ \beta_{s}\;\mathrm{if}\;{f(\beta }_{s}(c,d))<m_{P(c,d)}\\ \beta_{v}\;\mathrm{if}\;f(\beta_{v}\left(c,d\right))\leq m_{p(c,d)} \end{array}\right.$$

It is repeated for all three channels if the hue channel expected value is less than the mean probability. 

### 2.3. Spatial Convolution Neural Network

**Figure 2:**
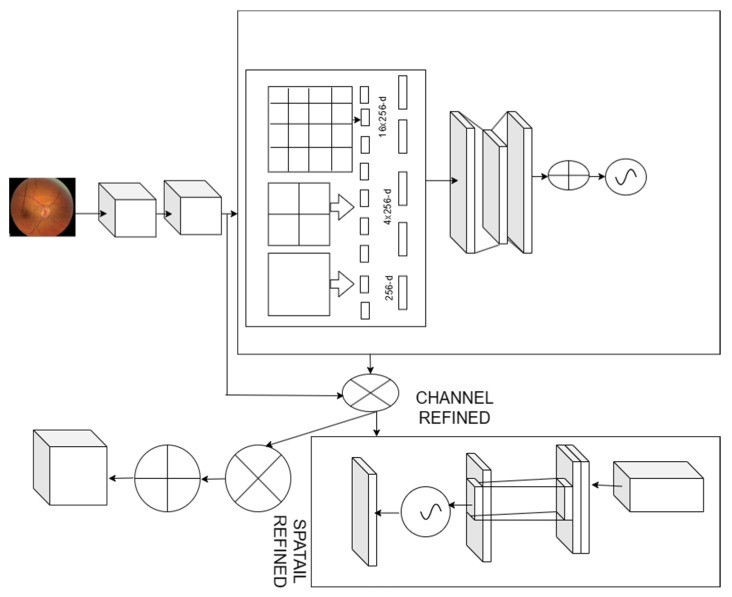
Spatial convolution attention module network.

We proposed exploiting the feature inter-channel connected to channel attention (Figure 2). It concentrates on the crucial details in the given fundus image. We reduce the spatial dimension of the input feature map to compute channel attention more accurately. We proposed spatial pooling as a solution to efficiently learn the distinctive properties of the target object. The intermediate feature map input $M\in A^{CX\;HXW}$, improved convolution and generates a 1D channel attention map sequentially $D_{C}\in A^{CX\;1X1}$. The complete improved convolution can be summarized:

(10) $$M^{\prime }=D_{C}\left(M\right)⊗M$$

(11) $$M^{\prime \prime }=D_{C}\left(M^{\prime }\right)⊗M^{\prime }$$

where ⊗ represents the element-wise multiplication. The channel attention values are transferred along the spatial dimension as the multiplication operation proceeds, and vice versa. To utilize the inter-channel relationship of features, we must create the channel attention mechanism. 

We considered each channel feature map to be the feature detector, and it concentrates on the provided input image. To assess the channel attention, consider the input feature map’s spatial dimension. We used the spatial pooling function to integrate the spatial information. The spatial pooling function collects all the distinctive objective features to obtain channel-wise attention. The spatial pooling function improves the model performance better than the remaining pooling functions.

The spatial layer provides input to the fully connected layers by pooling the features and producing outputs with specified lengths. Spatial pooling [29], an extension of the bag of words, collects the local features in the image’s segments from finer to coarser levels. Multilevel spatial bins are used in spatial pooling, which is resistant to object deformations and extracts the varying scales [30]. The number of bins remains constant regardless of the size of the image, since the spatial bins’ sizes are proportional to the size of the fundus image. To use the deep network for images of any size, we eventually replace the final pooling layer with spatial pooling. We combine the responses from each filter and the resulting KM dimensional vectors in each spatial bin, where D is the number of bins. We pool the features from various scales, after which we combine them. A feature map’s spatial data can be described using the descriptor $M_{S}^{c}$. The channel attention map $D_{C}\in A^{C\;X\;1\;X1}$ is created by sending the descriptors to a shared network. A single hidden layer in addition to a radial basis function network (RBFN) makes up the shared network. The hidden activation size has been assigned to $A^{C/eX\;1X1}$ where e is the reduction ratio. The element-wise summing is used to combine all of the feature vectors after applying each descriptor to the shared network. The improved convolution is represented as:

(12) $$D_{C}(M)=\sigma (RBF(P(M)))=\sigma \left(W_{1}\left(W_{0}(M_{s}^{C})\right)\right)$$

where $W_{0}\in A^{C/rXC}$ and $W_{1}\in A^{CXC/r}$. The weights of the RBF Wo and W1 are shared for each of the inputs, and W0 comes after the ReLU activation function. The inter-spatial relationship was used to create the spatial attention map, which concentrates on the map’s informative aspect. The informative regions are improved by applying pooling operations along the channel axis, and then the convolution layer is used to create the spatial attention map $D_{C\;}(M)\in A^{RXW}$, which specifies where to point out or suppress. The spatial attention is evaluated as:

(13) $$D_{C\;}\left(M\right)=\sigma \left(S^{9\times 9}\left(M_{S}^{c}\right)\right)$$

where σ represents the sigmoid function and $S^{9\times 9}$ depicts the convolution operation with a 9 × 9 filter.

For the given input image, the channel and spatial attention modules compute complementary attention. Arrange the two components either in parallel or in order. We found that a parallel arrangement produces a better outcome than a sequential one. Based on the experimental results, the channel first order is inferior to the spatial first order for the configuration of the parallel process.

We employ convolution in the spatial attention module with a kernel size of 9, and we organize the channel and spatial submodules sequentially. We select average- and max-pooling for both the channel and the spatial attention module. Furthermore, we examine the impact of varying kernel sizes at the subsequent convolution layer: 3, 7, and 9. When comparing various convolution kernel sizes, we discover that using a larger kernel size results in improved accuracy. Given this, we use the convolution layer with a large kernel size and the channel pooling approach to compute spatial attention. To sum up, the spatial attention module is based on average- and max-pooled data across the channel axis with a convolution kernel size of 9.

By utilizing the attention mechanism—that is, concentrating on significant traits and stifling lesser ones—we hope to boost the power of representation. We presented an enhanced convolutional block attention module in this research. We utilize our module to highlight significant features along the channel and spatial axes, as convolution methods extract informative features by combining cross-channel and spatial information. To do this, we apply the channel and spatial attention modules in turn, allowing each of them to learn “what” and “where” to focus on the channel and spatial axes, respectively. Consequently, by determining what information to emphasize or conceal, the proposed module effectively facilitates information flow inside the network.

From vessel segments, a new set of features is extracted, including textural features based on color, disc obscuration, and vascularity. These characteristics are applied to HR classification. To automatically capture all these changes, the proposed system extracts 24 features. The four feature groups that follow are texture, color, disc, and vascular features.

### 2.4. Optic Disk Segmentation

The optic disc is the bright, round region made up of the optic nerve fibers. The area of the eye fundus where the entire vascular tree appears is visible. The brightness and contrast of this area of the fundus image are different from other regions, which may affect the vessel’s primary characteristics and result in incorrect vessel categorization. To examine and classify the vascular anatomy, the optic disc region is, therefore, usually removed. Using k-means clustering, OD is identified and removed from the segmented retina image.

### 2.5. HR Classification

SVM/KNN classifiers use the 24 features that were covered in the previous section, which are taken from a collection of retinal images. For experiments, k-fold cross-validation with k = 10 is employed. We used support vector machines (SVM) and k nearest neighbors (KNN) classifiers to categorize the AV. The enhanced SVM and KNN classifiers used the extracted features to assess their performance. Algorithm 1 discusses the improved SVM and Algorithm 2 is about the improved KNN classifier. The classification results categorize the input segmented image into a specified class HR grade, which enhances the HR classification performance.

Upon feature extraction, the vessels are classified using the improved SVM. To assess the loss, the SVM first employs linear mapping on feature vectors to determine the score for each extracted feature. The enhanced SVM algorithm is presented in Algorithm 1.
**Algorithm 1** Enhanced SVM
**Input:** Feature Vector **Output:** HR classification ⮚    Initialize all values ⮚    for i = 1 to N    evaluate the loss    extract the lesions in the fundus images ⮚    End for     Evaluate the score vector for iterations i-1 to N         Evaluate the SVM score vector ⮚    End for         Evaluate output using the various weights.

**Algorithm 2** Enhanced KNN
**Input:** Feature Vector **Output:** HR classification ⮚    For each fundus image     Identify the lesion aspects ⮚    Create weak learners W_i_ ⮚    Determine whether the fundus image is healthy or diseased. ⮚    Integrate the results of the weak learners∑(W_i_) ⮚    Determine the training variance ⮚    Reweight the n-learners that are considered weak. ⮚    Identify the best weak learner with the least amount of training ⮚    Acquire classification outcomes

#### 2.5.1. SVM Variants

The kernel function is one of the most significant design decisions in SVM. Savas and Dovisu et al. [31] proposed the Gaussian kernel of SVM in a global navigation satellite system. Fine, medium, and coarse Gaussian kernel function SVM classifiers were used in the investigation. This outcome demonstrates how the performance of various Gaussian kernels—medium, coarse, or fine—varies based on the type of data to be analyzed, leading to various accuracy outcomes. Since the kernel selection has a big impact on SVM performance, it provides an implicit definition of the high-dimensional feature space’s structure, which is where the maximum edge hyperplane is located. The cubic polynomial kernel function, Gaussian kernel function, quadratic kernel function, and radial basis function are examples of frequently used kernel functions. However, the selection of kernel function pairs varies as well due to the various circumstances, as various kernels may exhibit varying performances.

Classification increases the accuracy of detection by classifying the veins and arteries. The blood vascular inaccurate classification is decreased by the suggested enhanced KNN linear classifier. Algorithm 2 provides the enhanced KNN linear classifier algorithm.

#### 2.5.2. KNN Variants

(i) Fine KNN: A nearest neighbor classifier that makes finely detailed distinctions between classes with the number of neighbors set to 1;
(ii) Medium KNN: A nearest neighbor classifier that makes fewer distinctions than a fine KNN with the number of neighbors set to 10;
(iii) Coarse KNN: The nearest neighbor classifier that makes coarse distinctions between classes, with the number of neighbors set to 100;
(iv) Cosine KNN: A nearest neighbor classifier that uses the cosine distance metric;
(v) Weighted KNN: The nearest neighbor classifier that uses distance weighting.

Cross-validation is a helpful method for assessing how well deep learning models work. In cross-validation, the dataset was split into training and test sets at random. The training set was used to create a model, and the test set was used to measure the accuracy of the model to determine how well it performed. The dataset was randomly split into k equal-sized subsets for k-fold cross-validation, with one serving as a test set and the others as training sets. To enable the use of every subset exactly once as a test set, cross-validation was performed k times. The mean model assessment scores computed over the k-test subsets were used to determine the model’s performance. In this research, we performed the 10-fold cross-validation. In the results we compared the 5-fold and 10-fold cross-validation and we identified a minute difference between these two folds.

### 2.6. Performance Metrics

This study uses the performance metrics, i.e., accuracy, precision, recall, f1 score, dice, and Jaccard coefficient for comparative analysis [32].

(14) $$\mathrm{Accuracy}=\frac{TP+TN}{TP+TN+FP+FN}$$

(15) $$\mathrm{Precision}=\frac{TP}{TP+FP}$$

(16) $$\mathrm{Recall}=\frac{TP}{TP+FN}$$

(17) $$F1-\mathrm{score}=\frac{1}{2}\Sigma \frac{Precision\times Recall}{Precision+Recall}$$

(18) $$\mathrm{Dice}\frac{2\times TP}{2\times TP+FN}$$

(19) $$\mathrm{Jaccard}\;\mathrm{Coefficient}\frac{TP}{TP+FN+FP}$$

## 3. Results

Retina images for hypertensive retinopathy were collected from distinct sources. To assess the hypertension severity, a five-class classification for hypertensive retinopathy has been developed in this study. Blood vessels are crucial for HR detection and grading because different HR stages cause structural changes in the vessels.

### 3.1. Segmentation Evaluation

**Figure 3:**
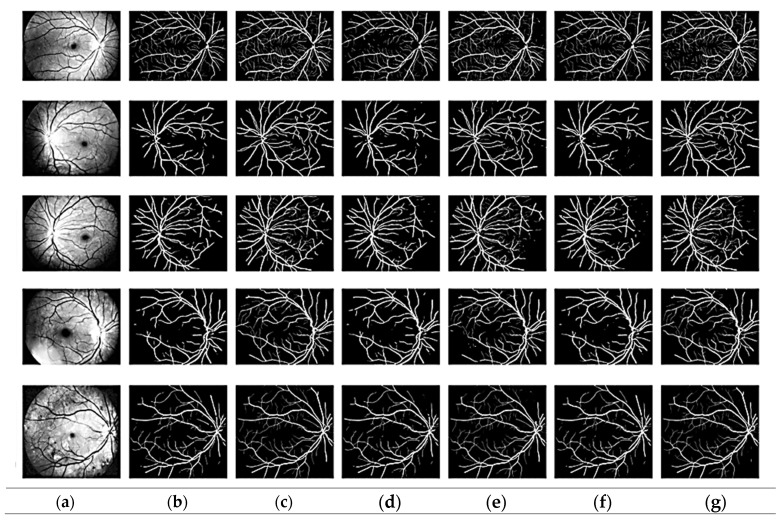
Segmentation output: (**a**) pre-processed, (**b**) ground truth, (**c**) proposed model, (**d**) GLUE [33], (**e**) SUNET, [34] (**f**) MUNET [35], (**g**) RESDUNET [36].

The suggested modified CNN model was used to extract features. Figure 3 shows that the suggested improved CNN can accurately segment the fundus image’s thickest blood vessel regions and other spots where a non-expert human eye would probably find it difficult to detect the presence of these vessels. Additionally, it should be emphasized that most existing models need help with focusing on the tiny blood arteries. The dataset includes ground truth images of manually segmented retinal vessels that can be used to evaluate the algorithms and approaches.

**Figure 4:**
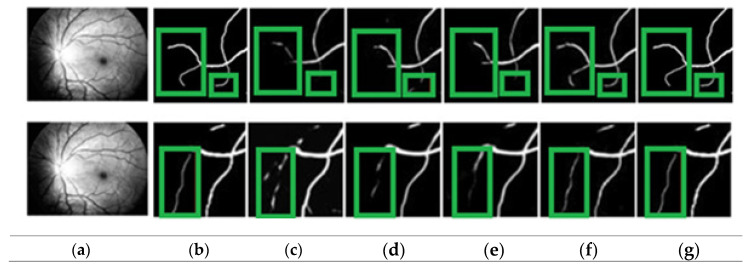
Identifying blood vessels: (**a**) pre-processed, (**b**) ground truth, (**c**) GLUE [33], (**d**) SUNET, [34], (**e**) MUNET, [35] (**f**) RESDUNET, [36] (**g**) proposed model.

Figure 4 shows how the proposed approach may more clearly identify thin blood vessels and ensure blood vessel connection. The proposed approach distinguishes between vascular and non-vascular pixels and better maintains vascular structure. From the visualization, it is clear that the proposed approach may fully assist the network in learning more feature data that ensures the connectivity of blood vessels. The variability results demonstrate that the proposed algorithm can produce significantly better segmentation, as seen by the segmentation’s predominant green box. As seen by the green box in Figure 4, these thin blood vessels were not properly segmented by the existing models. Therefore, it can be said that the proposed model outperforms state-of-the-art models in terms of vascular results. The comparison column offers useful details about how the actual output differs from the prediction. 

Locally enlarged versions of the original fundus images, the related truth values, the segmentation maps created using several additional techniques, and the proposed approach are all shown in Figure 4. The proposed paradigm makes it easier to identify thin blood vessels and guarantees blood–vascular connection. The proposed approach is more accurate at identifying the thin blood vessels.

We compared vessel recognition using the metrics dice. Most existing models provide results for vessel center line pixels, but the proposed approach assesses all vessel segments in the region of interest. In this study, we have considered the tiny capillaries in vessel classification. The vessel center line features exploit many colors for vessel characterization. Table 2 tabulates the comparison results of the state-of-the-art segmentation approaches to the proposed. For the glue model for the drive dataset, the accuracy is 96.92 and the precision is 86.37%; for the STARE dataset, the accuracy is 97.40% and the precision is 88.23%. In the SU-NET model for the drive dataset, the accuracy is 95.67% and precision is 83.12%, and for the CHASEDB1 dataset, the accuracy is 98.67% and the precision is 80.44%. For the MUNET model for the drive dataset, the accuracy is 96.92 and precision is 86.37%. For the Resdunet model, the accuracy is 96.92, the precision is 96.67%, and the recall is 96.8%.

**Figure 5:**
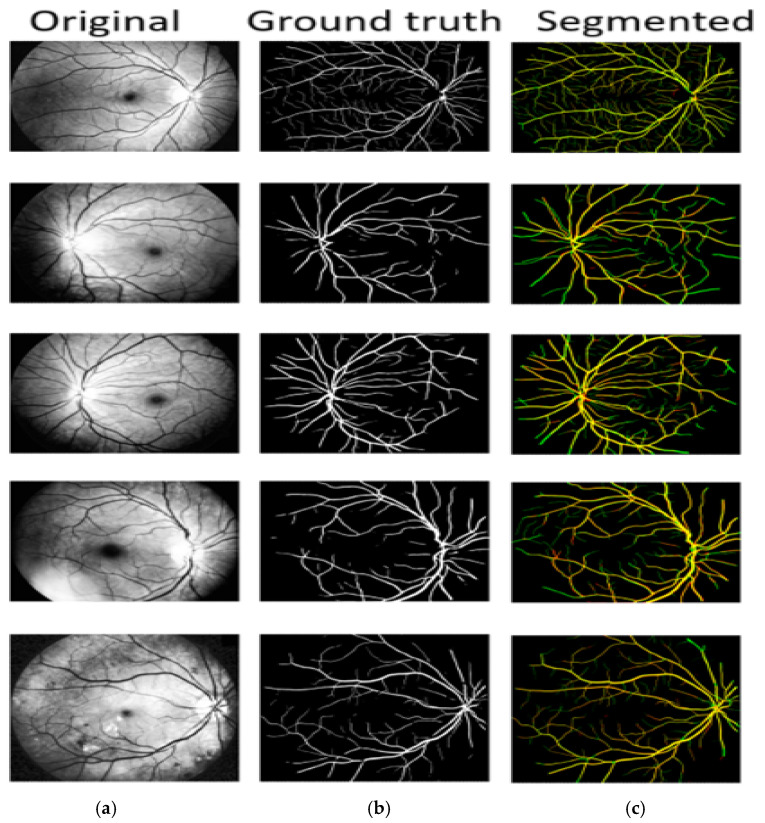
A/V classification: (**a**) original, (**b**) ground truth, (**c**) A/V classification.

Vascular patterns are extracted during HR self-diagnosis to further categorize them as arteries and veins. The area of interest is extracting potential retinal blood vessels, either vein or artery prospects. Arteriovenous crossings in vessel center lines are seen as one vessel segment in Figure 5. The cross-over locations in the original retrieved vessel map are eroded once the bifurcation and crossing-overs have been distinguished. Features are extracted from each prospective vessel segment afterward. The proposed model performs extremely well in regions with the thinnest blood vessels and arteries, as well as those closest to the optic nerve. The comparison column is a useful resource regarding the differences between the segmentation predicted by the proposed model and the actual data. True positive pixels are denoted by the yellow color, false positive pixels are characterized by three colors, and false negative pixels are denoted by the green color.

We utilize the INSPIREVR dataset to compare our approach with other cutting-edge techniques. Table 3 displays the indicators for the experimental evaluation. The accuracy for the INSPIREVR dataset that the proposed approach yielded are 99.04, respectively. Li et al. [22] et al. received the highest Sp scores among the existing models, Wang et al. [37] obtained the highest Acc among the existing models, and Mou et al. [38] obtained the greatest Se among the existing models. These values are significantly lower than proposed, and our Sp is just 1.05 higher than theirs. Our technique yields great results for accuracy, Sp, and Se. These findings demonstrate the superior vessel recognition and cross-database generalization capabilities of our model as compared to alternative approaches.

### 3.2. Classification Evaluation

This section deals with the classification of veins and arteries. Veins are distinguished by their low contrast, width, darkness, and color fluctuation. In comparison to arteries, the central reflex is smaller. Each centerline pixel’s features, as well as those of its surrounding pixels, have been retrieved. Both KNN and SVM classifiers receive this as input.

We used a dataset with five distinct classes in this study. A total of 20% of the data were split into test sets and the remaining 80% into training sets in order to generate the dataset models. The models’ performance was evaluated using the training dataset. A k-fold cross-validation (K = 1,2,…10) method was applied to assess the classifier performance. The best option when working with tiny datasets is to use k-fold cross-validation with a large k value. The cross-validation is a technique used to evaluate a predictive model’s performance. A particular kind of cross-validation called the 10-fold cross-validation method divides the dataset into ten subsets, or “folds.” Ten training iterations are required for the model, with the remaining nine folds serving as the training set and a separate fold serving as the test set. The primary benefit of applying cross-validation, and more especially 10-fold cross-validation, is that it contributes to a more trustworthy assessment of the generalization performance of the model. Compared to a single train–test split, it offers a more accurate estimation of the model’s performance on unknown data. Average performance metrics were acquired by running k runs of a k-fold cross validation, where k = 1, …, 10, to determine accuracy (%), precision (%), and recall (%).

**Figure 6:**
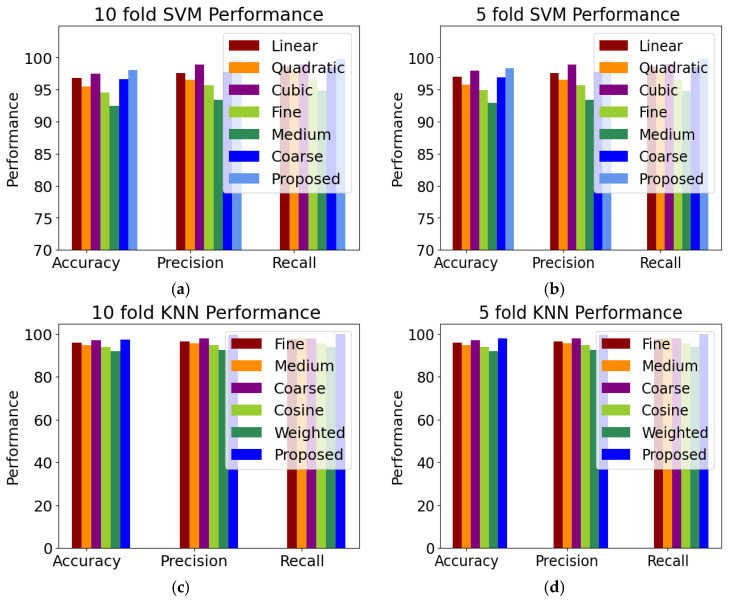
Comparison of classifiers (**a**) 10-fold SVM variants, (**b**) 5-fold SVM variants, (**c**) 10-fold KNN variants, and (**d**) 5-fold KNN variants.

The improved SVM and KNN classifier were further compared with the six variants of the SVM classifier, i.e., linear, quadratic, cubic, fine Gaussian, medium Gaussian, and coarse Gaussian. We also compared the improved KNN with the five variants of the KNN classifier. To categorize the HR grades, the improved feature vectors were input into the SVM and KNN classifier variations. Using five-fold cross-validation, Figure 6 displays the class-level outcomes for classifying the HR anomalies attained using SVM variants and KNN variants. We assessed the outcomes using several performance indicators, including accuracy, precision, recall, and F1 score. In this research, we proposed an improved KNN classifier and compared the proposed with the five variants of the KNN classifier, i.e., fine, medium, coarse, cosine, and weighted. The performance measures for the improved SVM are a sensitivity and specificity of 98.45% and 99.46%, respectively. After evaluating the performance of the classifiers, the improved SVM was determined to have the greatest accuracy. The medium Gaussian SVM has the lowest classification performance compared to the other SVM classifiers, with an accuracy of 84.96%. The ten-fold classification tabulated the comparison of the computational cost of the proposed highest accuracy of 98.99%, i.e., 0.27 higher than the five-fold classification accuracy. Figure 6 presents the comparison of the five-fold and ten-fold cross-validation. The KNN variants identify the closest neighbors by utilizing a similarity measure based on a distance matrix that was computed using a Euclidean method. The KNN classifier received the final, optimized feature vector to test the proposed approach’s effectiveness with several KNN classifiers. The proposed improved KNN classifier achieved an accuracy of 98.72%. The cosine KNN model obtained a slightly worse accuracy of 95.87% than the proposed model. More specifically, the coarse KNN model obtained the worst accuracy of 90.45%.

Table 4 tabulates the comparison of the classification results of the proposed model with the state-of-the-art models. Partial least square [43] achieved a classification accuracy of 96.05%, the SVM [44] achieved a classification accuracy of 97.46%, and the SVM radial basis function (SVMRBF) [45] achieved a classification accuracy of 98.72%; the proposed model achieved the highest accuracy of 98.99% using the improved SVM classifier.

### 3.3. Computational Cost Evaluation

We considered the pathologically diseased fundus images for evaluation of the proposed approach. State-of-the-art deep learning models are compared to the proposed model in terms of computation complexity. Table 5 tabulates the comparison of the computational cost of the proposed model with the state-of-the-art models. The pre-processing phase, which took 1.5 s, adjusted the brightness and eliminated noise from the input fundus images. Similarly, the proposed improved CNN system for feature extraction took 1.8 s. At the same time, the HR detection took only 1.6 s.

## 4. Discussion

The development of computational vision is a primary tool for automating disease diagnosis. With computer technology, all aspects unrelated to disease diagnosis are eliminated, reducing error risk and improving diagnostic precision [49]. This study proposes an automated computer vision system to identify and categorize HR. The pre-processing of fundus images is further improved and highlights the lesion and background regions. The blood vessels are segmented using the improved CNN to overcome the limitations of the existing models.

HR disease affects many people worldwide, although HR patients are unaware of this. An ophthalmologic examination of the patient’s eyes can identify HR and its severity. As a result of late-stage diagnosis of HR, the patient frequently loses vision or goes blind. Patients with HR should be careful to check their eyes periodically. Highly trained experts can only perform manual analyses, which are expensive and time-consuming. The determined features in this study are created so that the feature map is sufficiently precise to represent the most common characteristics of the blood vessels in order to recognize retinal blood vessels in unrestricted circumstances. Once the feature maps from an entire fundus image have been computed, features are pooled in subregions to create fixed-length representations for the fundus dataset’s training. The proposed model avoids repeatedly computing the convolution features, and it processes faster. The proposed system is lightweight, easily adaptable to any CNN design, incurs minimal overhead, and can be trained end-to-end alongside base CNN. For an accurate HR diagnosis, the retinal vasculature must be thoroughly examined. The segmentation using the proposed model detects tiny vessels. 

Using fundus photography, HR diagnoses can be made based on retinal images. A key factor for diagnosing hypertension is the diameter of retinal blood vessels, which is measured by the ratio of arterial blood vessels in the retina [50]. The progression of retinal diseases can be monitored by tracking changes in retinal blood vessels. The low ratio of the average diameter of arterial–venous (AVR) can be due to irregular vein width in HR [51,52]. Segmenting fundus images will lead to better treatment outcomes [53]. Due to the complex nature of manual segmentation and feature extraction, automatic segmentation is often recommended to address this issue and reduce error rate and time [54,55]. Some fundus image segmentation models were studied and applied to the considered dataset fundus images during this research. 

Improved CNN was also analyzed and applied, as well as its performance. The UNet [46] convolutional architecture is extensively used and recognized in domains like medicine where deep learning is utilized. Our key conclusions from training and testing this model are that it can perform well but it fails in the smallest blood vessel recognition. The visual transformer architecture (ViT) [47] and ConvMixer [48] are two other existing models taken into consideration. The ViT model is situated in the frame of attention mechanisms; we noticed slightly inferior outcomes than the initial UNet model, presumably caused by the small amount of data used to train the transformers. ConvMixer overcomes the limitations of transformers, performs better than UNet and ViTS, and produces outcomes that are just somewhat poorer. When only a few observations are available, there is no need to consistently employ large models to solve the fundus image segmentation challenges. Due to its increased robustness against overfitting and better generalization over unknown data, an insignificant architecture comprising the ideal components can outperform larger models.

By concentrating on the small vessels, the proposed approach enhanced the segmentation. A crucial stage in HR automated detection is segmenting tiny retinal blood vessels by the improved CNN model. The suggested model outperformed the most recent methods on the ODIR, INSPIREAVR, and VICAVR datasets, saving the accuracy attained, and obtaining precise segmentation results with the tiniest vessels. The outcomes demonstrate that the proposed approach performs outstandingly in the datasets’ most difficult parts, including the regions nearest to the optic nerve, where the bifurcation occurs, and where it intersects. The proposed approach produced exceptional results, demonstrating its ability to precisely identify arteries and blood vessels even in the thinnest parts.

Manually measuring retinal characteristics is a tedious procedure that takes time. Manual measurements frequently miss the minute signs of vascular disease and change. We compared the classification results to the various SVM and KNN variants. According to the results, the improved SVM attained the maximum accuracy for 10-fold classification. Although there are several automated models for HR detection, technologies that consider the entire fundus image for automatic HR diagnosis and grading are still necessary. The proposed model is helpful for ophthalmologists to study HR abnormalities, and experienced ophthalmologists can observe these abnormalities to diagnose various retinal illnesses. The proposed model helps to grade the HR accurately with low processing time.

## 5. Conclusions

An automated approach has been built to detect and classify HR. The improved Gaussian distribution was used to pre-process the fundus images and proposed a lightweight convolution model segment fundus images and later classify them using the improved SVM and the KNN classifiers. The proposed convolution module improves the retinal vessel segmentation that accurately recognizes the blood vessels. We compared the accuracy, precision, recall, and f1 score for each class, i.e., normal, mild, moderate, severe, and malignant, proposed with the existing models. The results show that the improved SVM has the highest classification accuracy of 98.99% and finishes the task in 160.4 s. The proposed model’s ability to quickly recognize various HR severity levels is significant when computational efficiency is critical. The results prove that the proposed system recognizes the vessels with high accuracy. The major limitation of this research is that we used the publicly available dataset but not the real-time dataset. We used the augmentation to artificially increase the training dataset. In the future, we will collect large real-time fundus images and smart phone images and implement the proposed model. We will identify other diseases like diabetic nephropathy, stroke, and rare cardiovascular diseases using fundus image.

## Figures and Tables

**Table 1:** HR symptoms.

Grade	Symptoms
Normal	Diagnosis of hypertension but no retinal abnormalities
Mild	Arteriolar narrowing
Moderate	Arteriolar narrowing with focal constriction
Severe	Retinal hemorrhage, focal and diffuse narrowing
Malignant	Hard exudates, optic disk edema

**Table 2:** Comparison of the segmentation results.

Model	Dataset	Dice	Jaccard Coefficient
GLUE [33]	DRIVE, STARE	81.45	75.47
SUNET [34]	DRIVE, CHASE DB1	81.74	77.46
MUNET [35]	Private	82.46	79.85
RESDUNET [36]	Private	77.36	78.47
Proposed	ODIR, INSPIREVR, VICAVR	83.27	83.93

**Table 3:** Segmentation performance comparison.

Model	Accuracy	Sensitivity	Specificity
Zhou et al. [39]	95.35	84.73	98.35
Xu et al. [40]	95.57	89.53	98.07
Wang et al. [37]	95.81	89.91	98.13
Li et al. [22]	95.73	98.35	98.38
Mouet al. [38]	95.53	81.54	97.57
Zhang et al. [41]	95.65	88.57	96.18
Liu et al. [42]	97.61	89.85	97.91
Proposed	99.37	98.75	99.56

**Table 4:** Classification comparison results.

Model	Accuracy	Sensitivity	Specificity
Partial least square [43]	96.05	77.76	98.32
SVM [44]	97.46	83.78	83.08
SVMRBF [45]	98.58	81.86	81.95
Proposed (improved SVM)	98.99	98.45	99.46
Proposed (improved KNN)	98.72	98.26	99.39

**Table 5:** Evaluation of the average processing time.

Model	Pre-Processing (s)	Segmentation	Training (s)	Prediction (s)	Overall (s)
GLUE [32]	3.8	4.7	191.5	3.5	203.5
SUNET [34]	3.5	4.2	189.8	3.2	200.7
MUNET [35]	3.5	3.5	189.4	2.5	198.9
RESDUNET [36]	2.6	3.2	174.3	2.2	182.3
Unet [46]	2.4	2.1	168.4	1.9	174.8
ViT [47]	1.9	1.9	158.5	1.8	164.1
ConvMixer [48]	1.8	1.9	156.9	1.7	162.3
Proposed	1.5	1.8	155.5	1.6	160.4

## Data Availability

The data presented in this study are available on request from the corresponding authors.
